# Clinical assessment, investigation, diagnosis and initial management of cerebral visual impairment: a consensus practice guide

**DOI:** 10.1038/s41433-022-02261-6

**Published:** 2022-10-18

**Authors:** Rachel Fiona Pilling, Louise Allen, Richard Bowman, John Ravenscroft, Kathryn J Saunders, Cathy Williams

**Affiliations:** 1 University of Bradford, Bradford, England; 2 Department of Ophthalmology, Bradford Teaching Hospitals, Bradford, UK; 3 University of Liverpool, Liverpool, England; 4 Great Ormond Street Hospital, London, England; 5 University of Edinburgh, Edinburgh, UK; 6 Ulster University, Coleraine, UK; 7 University of Bristol, Bristol, England

**Keywords:** Eye manifestations, Risk factors, Education

## Abstract

Cerebral Visual Impairment (CVI) is a common condition in the UK. Patients with conditions associated with CVI are frequently seen in paediatric ophthalmology clinics offering eye care professionals an opportunity to identify children proactively. In most cases CVI occurs as part of a neurodevelopmental condition or as a feature of multiple and complex disabilities. However, CVI can also be seen in children with apparently typical development. In some cases, high contrast visual acuity is normal and in other cases severely impaired. As such, identification of CVI requires evaluation of aspects of visual performance beyond high contrast acuity and consideration that visual function of those with CVI may fluctuate. Few paediatric ophthalmologists have received formal training in CVI. The detection and diagnosis of CVI varies across the UK and patients report hugely different experiences. A diagnosis of CVI is made based on professional clinical judgement and it is recognised that individual perspectives and local practice in the specific methodologies of assessment will vary. A systematic review and survey of professionals is underway to attempt to reach agreement on diagnostic criteria. Nonetheless, established pathways and published protocols can offer guidance on how a paediatric ophthalmology service can approach assessment of the child with suspected CVI. The purpose of this paper is to present a summary of research and clinical practice methods for detecting and diagnosing CVI in a paediatric ophthalmology outpatient setting. It represents current understanding of the topic and acknowledges the evolving nature of both practice and the evidence-base. A rapid literature review was undertaken to identify articles relating to clinical investigation of children with CVI. A focus group of QTVI and subject matter experts from sight loss charities was undertaken to address areas which were not covered by the literature review.

## Abstract (zh)

脑源性视力障碍(CVI)是英国的常见疾病。患有CVI相关疾病的患者经常在儿科眼科诊所就诊, 为眼科护理专业人员提供了主动识别儿童CVI的机会。多数情况下, CVI为神经发育疾病的一部分, 或作为多种复杂残疾的一种临床体征。然而, CVI也可见于发育正常的儿童。在某些情况下, 高对比度视力可以正常, 而在其他情况下则严重受损。因此, CVI的识别需要评价高对比度视力以外的视觉表现方面, 并考虑CVI患者的视觉功能波动的可能性。很少有儿科眼科医生接受过CVI的正式培训。CVI 的检测和诊断在英国各不相同, 患者报告的体验差异很大。CVI的诊断是基于专业的临床判断, 并且需认识到特定评估方法中的个体观点和当地实践有所不同。目前正在对专业人员进行系统审核和调查, 以试图就诊断标准达成一致。尽管如此, 已确立的途径和已发表的方案可为针对如何处理疑似CVI儿童的儿科眼科治疗评估提供指导。

本文旨在对在儿科眼科门诊检测和诊断CVI的研究和临床实践方法进行总结。它代表了目前对这一专题的理解, 并承认了实践和证据基础的演变特性。本文进行了快速文献回顾, 以识别与CVI儿童临床研究相关的文章。我们成立了聚焦于QTVI 的专家小组并组织关心视力丧失的慈善机构专家以解决文献综述未涵盖的领域。

## Introduction

Cerebral Visual Impairment (CVI) is a common condition in the UK. Patients with conditions associated with CVI are frequently seen in paediatric ophthalmology clinics offering opportunity eye health professionals an opportunity to identify children proactively. In most cases CVI occurs as part of a neurodevelopmental condition or as a feature of multiple and complex disabilities. However, CVI can also be seen in children with apparently typical development. In some cases, high contrast visual acuity is normal and in other cases severely impaired. As such, identification of CVI requires evaluation of aspects of visual performance beyond high contrast acuity and consideration that visual function of patients with CVI may fluctuate. CVI has not historically been included in Royal College of Ophthalmologists trainee curriculum and as such trainees lack awareness of the condition. Few paediatric ophthalmologists have received formal training in CVI [1]. For these reasons, the detection and diagnosis of CVI varies across the UK and patients report hugely different experiences.

The purpose of this paper is to present a summary of research and clinical practice methods for detecting and diagnosing CVI in a paediatric ophthalmology outpatient setting (Box 1). It represents current understanding of the topic and acknowledges the evolving nature of both. A concise summary of this document has been reviewed by members of the UK CVI specialist interest group (eye health professionals from the UK).

Box 1: Summary of questions arising from multidisciplinary consultation What history, examination and investigations should be undertaken for a child attending an NHS paediatric ophthalmology clinic when referred with suspicion of cerebral visual impairment?

What findings would constitute a diagnosis of cerebral visual impairment?

What should be written in a report to the healthcare and education team?

What support/advice/signposting/onward referral should be offered by a general paediatric ophthalmology team?

### What is cerebral visual impairment?

Cerebral visual Impairment (CVI) is an umbrella term which encompasses a wide range of brain-related visual problems [2]. Cerebral Visual Impairment is the commonest cause of visual impairment in children in the developed nations [3–6]. Whilst there is broad consensus around the features of CVI and the concept of visual difficulties not explainable by ocular findings [7], there are as yet no agreed diagnostic criteria or thresholds for diagnosis.

Some authorities regard impairments of visual acuity or visual field (not due to ocular or optic nerve pathology) as necessary for a diagnosis of CVI, while others regard any brain-based visual deficit as potentially part of CVI [8]. For the purposes of this document, we take the more inclusive final option, while awaiting agreement on national and international guidelines.

There is often no cure for the features of cerebral visual impairment: rather the impact of visual dysfunction is ameliorated by adopting strategies and modifications to the child’s environment tailored to the child’s unique combination of difficulties [9–11].

### Current consensus for definition of CVI [7]

A verifiable visual dysfunction which cannot be attributed to disorders of the anterior visual pathways or any potentially co-occurring ocular impairment.

A diagnosis of CVI is made based on professional clinical judgement and it is recognised that individual perspectives and local practice in the specific methodologies of assessment will vary [8]. A multidisciplinary working group in Europe has been created to carry out a systematic review and survey professionals to reach agreement on diagnostic criteria. Nonetheless, established pathways and published protocols can offer guidance on how a paediatric ophthalmology service can approach assessment of the child with suspected CVI.

### Epidemiology

Cerebral Visual Impairment is the most common cause of registrable visual impairment in the UK [12]. CVI has been reported as affecting 20–90% children with common neurodevelopmental conditions (prematurity, cerebral palsy, hypoxic ischaemic encephalopathy, hydrocephalus, meningitis, Down’s syndrome [3–6, 13]) and children with special educational needs are 28 times more likely to encounter vision problems than typically developed children [14]. The CVI Project (www.thecviproject.co.uk) found that at least 3% of children in mainstream school—equating to one child in every classroom—had CVI-related visual difficulties [15]. This places CVI and related vision problems on a par with amblyopia as a common childhood vision disorder [16].

Broadly speaking, children with cerebral visual impairment have been reported as having one of three profiles:

i. Those with marked/moderate developmental delay and impaired visual acuity as well other variable visual deficits.
ii. Those with mild or no developmental delay, normal or near normal visual acuity and impaired oculomotor and vision processing abilities.
iii. Those with mixed characteristics [17].
  There is emerging a fourth group.
iv. Those who have functioned well and appeared asymptomatic until the point of decompensation.

Regardless of which group the child lies in or profile they present with, each will have significant visual difficulties affecting daily life, education and independence.

### What is the role of the ophthalmologist in the identification and diagnosis of cerebral visual impairment?

Ophthalmologists play an important role in both exploring the visual development concerns of parents, teachers and other health professionals and making a timely formal diagnosis to enable children to access services and/or support.

**Fig. 1:**
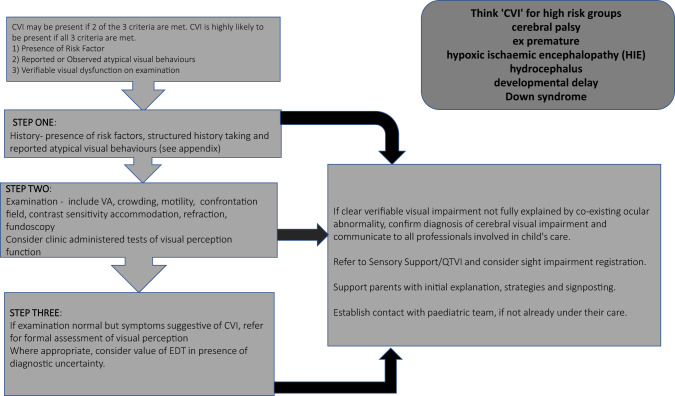
Stepwise approach to the diagnosis of paediatric cerebral-visual impairment.

**Fig. 2:**
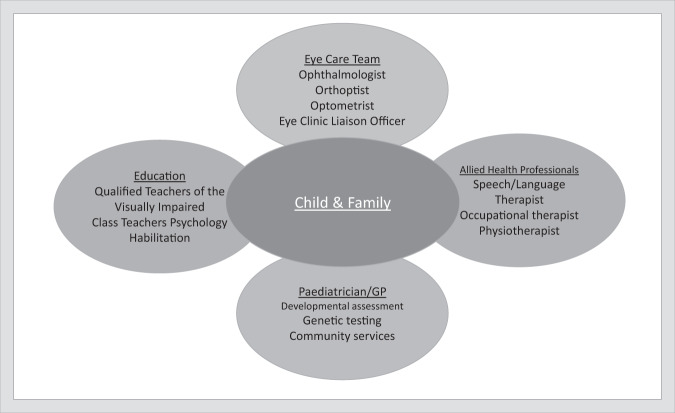
The team around the child: agencies to involve with any child diagnosed with CVI. Multi-agency invovement in a child with suspected or confirmed Cerebral Visual Impairment.

Figure 1 outlines the assessment and initial management of a child referred with suspected CVI. It is acknowledged that existing services may have alternative approaches. Collaborative working between paediatric, ophthalmology, developmental support services and education will refine local arrangements (Fig. 2).

Whilst it is beyond the scope of this document to specify a model of care a general paediatric ophthalmology department can be expected to support the diagnosis of CVI by:

- Excluding and/or managing co-existing ocular conditions (e.g. optic disc anomalies, structural anomalies such as cataract, coloboma), refractive error and accommodation dysfunction.
- Establishing by history-taking risk factors for, and symptoms of, CVI to enable proactive detection of visual dysfunction or behaviours indicative of such.
- Considering involvement of the whole healthcare team in particular paediatric and developmental disability teams, in establishing associated conditions.

### How is cerebral-visual impairment diagnosed?

There are three main elements to the diagnosis of CVI (see Tables 1, 2). Where there is a positive finding in all three areas, it is probable that CVI is present. Where there is a positive finding in two of the three elements, CVI should be considered, and further evidence to support or exclude a diagnosis sought from parents or other professionals.

**Table 1:** Cerebral-visual impairment diagnostic elements.

1. Developmental anomaly/positive risk factor for CVI	e.g. ex premature, cerebral palsy, Down’s syndrome, hypoxic ischaemic encephalopathy, hydrocephalus, developmental delay.
2. Externally observed or reported symptoms of CVI and/or atypical visual function	e.g. Unexplained failure to progress at school; difficulty finding objects; difficulty with complexity; clumsiness; difficulty recognising people, shapes, places; difficulty with moving objects; eccentric visual behaviours; may include information from inventories/questionnaires.
3. CVI-related visual dysfunction elicited on examination in any combination of areas below (Table 2).	e.g. Traditional acuity tests, crowding test, contrast sensitivity or low contrast acuity; confrontation field, dynamic retinoscopy, refraction, oculomotor function (including pursuits, saccades), simple clinic-based perceptual tests.

**Table 2:** Commonly encountered cerebral-visual impairment related visual dysfunctions.

Lower visual function impairment	Higher visual function impairment ventral stream	Higher visual function impairment dorsal stream
Reduced binocular visual acuity (in particular with crowded testing) not due to refractive error at distance or near Abnormal fixation Visual field deficit/inattention Oculomotor impairment (in particular jerky smooth pursuit, inaccurate saccades)	Visual memory/recognition (impaired ability to name shapes, objects, letters, recognise faces, or facial gesture) Route finding/orientation (impaired ability to ‘map’ their world or a room in order to orient effectively through it)	Impaired ability to notice more than one object at a time (simultanagnosia) Movement perception (impaired ability to see moving objects OR impaired ability to see objects unless they are moving) Visual guided movement (impaired ability to reach for/locate objects/navigate steps/kerbs)

Children with CVI may have significant visual difficulties affecting daily life, education, and independence. The spectrum of dysfunction and presentations requires a curious, proactive, and flexible approach to history taking, examination and diagnosis which will differ for each child.

#### Diagnosis: history taking

History taking is an essential first step. The past medical history may elicit events related to birth injury, hydrocephalus, cerebral palsy, prematurity, meningitis and neurodevelopmental abnormalities (structural or metabolic). The use of inventories/questionnaires/structure history taking in eliciting descriptions of visual behaviours can be useful to direct a testing or clinical examination strategy and are considered in Section 6.

#### Diagnosis: documenting areas of visual difficulty

The broad features of CVI relate to visual acuity, viewing behaviour (fixation), supranuclear eye movements (pursuits or saccades) or field impairment. If any one of these is present in a child with other developmental delay, the child will need support to use their vision to access education. It is helpful for the family to have formal recognition and documentation of visual dysfunction, whether or not the individual ophthalmologist determines this to have met the threshold for a diagnosis of CVI. Raising suspicions of CVI at as early a stage in development as possible enables the child to access support and develop strategies or environmental adaptations to use their vision effectively. There should not be a delay in diagnosis while waiting for the child’s development to allow for formal testing of acuity, visual fields or visuo-perceptual status (Visual inattention encompasses a wide variety of atypical visual behaviours, including visual avoidance, eccentric fixation, poor fixation, light gazing and visual stasis (being unable to look away from an object)).

#### Diagnosis: formal and informal testing

Deficits in higher visual functions can be elicited from a history or by informal or formal testing. Visuocognitive/visuoperceptual assessments are more commonly performed by occupational therapists, psychologists and other allied professionals. Basic (low cognitive demand) tests of visuoperceptual/visuocognitive skills are emerging. These tests can be performed by eye departments and/or teachers of the visually impaired and normative data are available [18] (see Appendix 1). Ophthalmologists with experience of these tools may choose to use them to confirm a diagnosis of CVI and highlight the specific visuoperceptual domains within which a child demonstrates impairment and therefore requires further assessment by relevant professionals as well as relevant support.

### Examination and visual assessment

The overriding principle to hold central is that not every child needs every test. A testing strategy should be flexible and inclusive. The skill lies in adapting testing techniques to the child’s developmental ability rather than delaying a diagnosis until the child can engage with specific tests. This is similar to other areas of paediatric ophthalmology where the aim is to establish the most important things first and be prepared to repeat assessments if needed for confirmation.

The visual assessment required to make or exclude a diagnosis of CVI is in many cases within the remit of a general ophthalmology service using existing tools and equipment. Appendix 1 identifies the validated tests and tools which might be utilised.

#### The role of inventories/questionnaires in the diagnosis and management of CVI

Structured questions relating to the child’s visual function can be helpful in eliciting signs and symptoms of CVI and may supplement general history taking. The child themselves will perceive their vision as ‘normal’ to them and is often unable to describe what is ‘unusual’ about their vision [19].

Whilst not essential for diagnosis, structured inventories/questionnaires can be useful in devising an examination strategy for the individual child.

Many inventories and questionnaires have been published as validated instruments [20–31], however no single tool has been shown to be reliable in the diagnosis of CVI [8]. Studies have shown that stand-alone questionnaires lack the sensitivity required for reliable diagnosis and their use as broad screening tools is likely to over-diagnose [32, 33]. Any particular behaviour, such as failing to find someone in a crowd, may have a variety of visual or non-visual causes. However, inventories/questionnaires are a useful prompt for parent and practitioner discussions and a way of identifying key problem areas in day-to-day life. A table of validated tools is provided in Appendix 2.

The ‘Insight’ inventory has been shown to be an effective tool in estimating visual-perceptual difficulties without formal testing [21]. When linked to targeted strategies to support the child in using their vision effectively, its use can facilitate improvement in function vision and quality of life [20, 21].

### The role of investigations in the diagnosis of cerebral visual impairment

CVI is a clinical diagnosis made based on history taking, visual dysfunction (on examination or in the presence of behaviour suggestive of visual dysfunction) and the presence of an underlying risk factor. Therefore, formal investigations are often not required in the diagnosis of CVI. However, they can be useful when there is diagnostic uncertainty or to identify co-existing ocular/neurological pathology, particularly optic neuropathy which may be secondary to lesions in the primary visual pathway.

#### Neuroimaging

Most children presenting with developmental abnormalities have already had neuroimaging and reviewing a previous scan can be useful. However, around a third of patients with CVI have no visible abnormality on routine structural imaging [5, 19]. The risk from general anaesthesia should be balanced against any potential benefit to the patient from undergoing neuroimaging. The evidence correlating visual dysfunction and CVI with MRI findings is weak [34] and imaging should not be seen as a primary investigation unless it is going to directly alter the management of the patient [35].

#### Electrodiagnostic Testing (EDT)

Electrodiagnostic tests are not essential for the diagnosis of CVI [35]. They may have a role in the identification of retinal or optic nerve dysfunction where co-existing ophthalmic (e.g. nystagmus, high refractive error) and/or medical diagnoses (e.g. metabolic or genetic conditions) have a known association with retinal dystrophy. In the presence of normal acuity VEPs are unlikely to demonstrate any abnormalities [36].

#### Ocular Coherence Tomography (OCT)

OCT of the optic disc is emerging as a tool within the diagnosis of CVI, particularly for children who cannot perform standard perimetry [37]. Periventricular leucomalacia (PVL) related disc cupping is a common finding [38–40]. Focal thinning of the ganglion cell layer has been found to be present in areas corresponding to visual field defects and sector asymmetry has been shown to be predictive of a visual field defect [41]. In the absence of sector asymmetry, visual field is likely to be normal or globally constricted [37].

#### Formal perimetry

Formal visual field assessment is not essential in the diagnosis of CVI; indeed, for many children it will be beyond their developmental abilities to perform such test. Perimetry may be useful in higher functioning children to demonstrate field loss and support sight impairment registration.

#### Visuo-perceptual or Psychometric testing

Visuo-perceptual testing is important in profiling of children with CVI to fully uncover the breadth of visual dysfunction a child may be experiencing [5, 9, 19, 42–46]. Visuo-perceptual tests often require good central visual acuity and well developed communication, motor and cognitive ability in order for results to be meaningful. For children with cerebral-visual impairment in the context of marked developmental delay, the current tests are not accessible.

Psychometric tests do not form part of the core visual assessment; however it is important that ophthalmologists arrange or suggest referral to professionals (eg Neurodevelopmental Paediatricians, psychologists, occupational therapists or QTVIs) in the local area who have appropriate expertise. Some children with normal acuity and field will require such testing in order to demonstrate verifiable visual dysfunction to fulfil the diagnostic criteria and receive a confirmed diagnosis of CVI. It is also an ophthalmologist’s role to liaise with multidisciplinary teams to establish a holistic picture of the child’s wider neurological, physical and sensory status when exploring a diagnosis of CVI.

For those children who can participate, specialist ophthalmology/MDT clinics may wish to consider incorporating tests shown in table in Appendix 2.

### Management of Children with CVI

Parents commonly cite a lack of support, signposting and information at diagnosis as a key barrier to them accessing services to support their children [11, 47]. Whilst each individual child will need a bespoke support package, simple adaptations to educational materials (such as enlarged font and/or fewer items per page) and environments (such as reducing clutter and/or considering area of best visual attention) help a lot of children [48]. Appendix 3 offers signposting to strategies which health, education and parents may find useful.

Ophthalmologists are well placed to offer initial explanations, advice, and referral on to other health, education, and social care professionals. It is essential that key ocular issues which may not be apparent to other professionals supporting the child, such as refractive errors, hypoaccommodation, impaired saccades and pursuits and field loss, are identified, managed and communicated effectively.

Whilst it is beyond the scope of this document to specify a model of care, where CVI is identified or suspected, ophthalmologists and their teams might undertake the following steps:

i. Acknowledge the presence of CVI and provide an explanation of the nature of the child’s visual disability, including signposting information.
ii. Manage refractive error including bifocals for children with poor accommodative function.
iii. Offer information on initial strategies based on clinical findings (e.g. field of attention, difficulties tracking objects, difficulty with clutter).
iv. Contribute to effective report writing encompassing a description of the child’s visual function, beyond a simple acuity measurement and share this report with parents and the wider team supporting the child.
v. Arrange referral to education service (sensory support team, QTVI, habilitation services).
vi. Consider sight impairment registration, (or notification to VINCYP if in Scotland) regardless of visual acuity, if the child is judged to be significantly disadvantaged by their visual function.
vii. Refer to the Eye Clinic Liaison Officer (where available) and signpost charity/support organisations (Appendix 4).
viii. Where appropriate, to arrange onward referral for further assessment and support (e.g. sensory services, occupational therapy, psychology, low vision clinic).

#### Prognosis

It is important to explain to parents that while there is no ‘cure’ for CVI, it is not true to say that ‘nothing can be done’. Improvements in function can be seen over time in some cases, and support from education, habilitation, occupational therapy, and family can be transformative as children learn strategies to maximise the efficiency of their vision, finding ways to ‘make it easier to see’ [48]. It should be acknowledged that as children mature, the visual burden or expectations on them increase. This often brings an apparent drop in function which can be managed by exploring and adopting new strategies for emerging situations and needs.

### Effective report writing in CVI

It is important that eye health professionals provide accessible and meaningful information about the child’s visual strengths and weaknesses to education teams to facilitate provision of support [6, 11, 14, 22, 43, 47, 49, 50]. Inclusion of any visual impairment within a child’s Education Health and Care Plan (EHCP) or equivalent is crucial but often overlooked.

The following key phrases have been suggested by teachers of the visually impaired (QTVI) as being helpful to sensory service providers.

i. Brief explanations of what CVI is (*e.g. ‘The child’s brain has difficulty processing the information it receives from the eyes in order to make sense of the world around them’)*.
ii. Inclusion of the diagnostic term ‘Cerebral Visual Impairment’ where the ophthalmologist is confident this is warranted.
iii. A statement indicating the need for support to access education. ‘*This child’s visual dysfunction will impact on their ability to access education/develop social skills/mobilise safely and independently including road safety/communicate effectively*’.
iv. Recommendation that visual impairment be included in the child’s EHCP or equivalent.

Elements of the visual assessment important to include in a report are shown below.

- Visual acuity, or object size and distance at which it is seen
- Size and contrast of print the child can easily access
- Areas of visual field inattention e.g. inferior loss, or hemianopia or global constriction
- Ocular motility—smooth pursuits and saccades—ability to track objects, locate new objects
- Duration of/Fluctuation in visual attention
- Any abnormal head posture adopted to maximise vision e.g. looks to side of motility problems etc
- Contrast sensitivity
- Requirement for glasses and when they should be used
- Information from observation or history regarding visual clutter, visual memory, shape, place, face recognition
- Results from questionnaires/inventories which highlight strategies which match the child’s areas of visual strength/weakness.

## Appendix 1: Visual assessment tools for children with and without verbal/motor skills

**Table:**

Visual function	Test for verbal/motor children	Test for pre-verbal/pre-motor children
Visual acuity: Near and Distance	logMAR letter tests (e.g. Keeler crowded letter test, Sonksen logMAR letters) LogMAR picture tests (e.g. Kay picture test, Lea symbols)	Cardiff acuity test Teller/Keeler grating acuity test Questions 1–11 of PreViAs [30]
Visual attention: Duration distance and object size	By observation	Bradford Visual Function Box [6, 51] Mirror test [26, 52] Puppet Face Neonatal Assessment Visual European Grid [26]
Crowding ratio [33, 53] for Near and Distance (difference between acuity for single optotypes vs. crowded optotypes with both eyes open).	logMAR letter tests (e.g. Keeler crowded letter test, Sonksen logMAR letters) LogMAR picture tests (e.g. Kay picture test, Lea symbols)	N/A
Contrast Sensitivity or Low Contrast Acuity	Low contrast letter/picture charts (Lea symbols, ETDRS)	Hiding Heidi [54] Cardiff Contrast test [55]
Confrontation visual fields, including inferior visual field	Using appropriate object size and mindful of visual field inattention: horizontal and vertical	Using appropriate object size and mindful of visual field inattention: horizontal and vertical
Simultanagnosia	Ability to detect a ‘new’ object in a different area of visual field	
Pupil reactions		
Pursuit and saccades	Using appropriate object size and mindful of visual field inattention; horizontal and vertical	
Accommodation	Dynamic retinoscopy	
Object orientation	Lea mailbox [56] (for children over developmental age 4 years)	
Ability to match shapes visually	Lea rectangles [18, 56] (for children over developmental age 6 years)	Lea 3D puzzle test
Oculo-motor coordination (Reach and Grab)	Using a suitable size object based on visual acuity. Observing accuracy, speed and eye/head alignment during task.	
Ability to detect moving objects	CVIT 3–6 [42, 57]	By observation

Visuo-perceptual tests

**Table:**

**Tool**	**Notes/ref (e.g. age range, area of function assessed)**
Child Visual Impairment Test for 3–6 year olds (CVIT 3–6) [42]	12-min (tablet/online interaction visual tasks)
Beery Visual-Motor Integration test (VMI)	3 tasks
L94 visual perception battery	8 tasks
Test of Visual Perceptual Skills—Revised (TVPS-R)	7 subscales
Developmental Test of Visual Perception (DTVP)	5 subscales

## Appendix 2: Cerebral-visual impairment inventories/questionnaires

**Table:**

Tool	Notes/reference
Insight/Visual Skills Inventory	[21, 23] http://www.ctevh.org/Conf2017/Workshops/407/407%20-%20Insight.html 52 item questionnaire with links to strategies
PreVias	[30] Tool for children under 12 months
TeACH	https://www.teachcvi.net/ 3 questionnaires for children of differing age (6+, under 6) or motor abilities (version for non-ambulant children). [28]
Flemish CVI questionnaire	[29] https://cdn-links.lww.com/permalink/coop/a/coop_2012_05_31_lehman_656_sdc1.pdf 46 item tool designed to draw out behaviours associated with CVI

## Appendix 3: Strategies to support children with CVI

Insight: Approaches for Visual Perceptual Difficulties [22] http://www.ctevh.org/Conf2017/Workshops/407/407%20-%20Insight.html.

Make It Easier To See [48] https://makeiteasiertosee.co.uk/.

Ulster University CVI Strategies

https://www.ulster.ac.uk/research/topic/biomedical-sciences/research/optometry-and-vision-science/vision-resources/professionals/cerebral-visual-impairment-assessment.

Colour Tents [58] https://cvisociety.org.uk/news.php?cat_id=250.

## Appendix 4: UK CVI charities and patient support organisations

www.cvisociety.org.

www.cviscotland.org.
